# Urban social environment and low birth weight in 360 Latin American cities

**DOI:** 10.1186/s12889-021-10886-7

**Published:** 2021-04-26

**Authors:** Santiago Rodríguez López, Natalia Tumas, Ana Ortigoza, Amélia Augusta de Lima Friche, Ana V. Diez-Roux

**Affiliations:** 1 Centro de Investigaciones y Estudios sobre Cultura y Sociedad, Consejo Nacional de Investigaciones Científicas y Técnicas y Universidad Nacional de Córdoba, Córdoba, Argentina; 2 Facultad de Ciencias Exactas, Físicas y Naturales, Universidad Nacional de Córdoba, Córdoba, Argentina; 3 Facultad de Ciencias de la Salud, Universidad Católica de Córdoba, Córdoba, Argentina; 4 Facultad de Ciencias Médicas, Universidad Nacional de Córdoba, Córdoba, Argentina; 5 Dornsife School of Public Health, Drexel University, Philadelphia, USA; 6 Facultade de Medicina, Universidade Federal de Minas Gerais, Belo Horizonte, Brazil; 7 Department of Epidemiology and Biostatistics, Dornsife School of Public Health, Drexel University, Philadelphia, USA

**Keywords:** Low birth weight, Social environment, Maternal education, Urban, Latin America

## Abstract

**Objective:**

Using data compiled by the SALURBAL project (Urban Health in Latin America; ‘Salud Urbana en América Latina’) we quantified variability in low birth weight (LBW) across cities in Latin America, and evaluated the associations of socio-economic characteristics at various levels (maternal, sub-city and city) with the prevalence of LBW.

**Methods:**

The sample included 8 countries, 360 cities, 1321 administrative areas within cities (sub-city units) and birth registers of more than 4.5 million births for the year 2014. We linked maternal education from birth registers to data on socioeconomic characteristics of sub-cities and cities using the closest available national population census in each country. We applied linear and Poisson random-intercept multilevel models for aggregated data.

**Results:**

The median prevalence of city LBW by country ranged from a high of 13% in Guatemala to a low of 5% in Peru (median across all cities was 7.8%). Most of the LBW variability across sub-cities was between countries, but there were also significant proportions between cities within a country, and within cities. Low maternal education was associated with higher prevalence of LBW (Prevalence rate ratios (PRR) for less than primary vs. completed secondary or more 1.12 95% CI 1.10, 1.13) in the fully adjusted model. In contrast, higher sub-city education and a better city social environment index were independently associated with higher LBW prevalence after adjustment for maternal education and age, city population size and city gross domestic product (PRR 1.04 95% CI 1.03, 1.04 per SD higher sub-city education and PRR 1.02 95% CI 1.00, 1.04 per SD higher SEI). Larger city size was associated with a higher prevalence of LBW (PRR 1.06; 95% CI 1.01, 1.12).

**Conclusion:**

Our findings highlight the presence of heterogeneity in the distribution of LBW and the importance of maternal education, local and broader social environments in shaping LBW in urban settings of Latin America. Implementing context-sensitive interventions guided to improve women’s education is recommended to tackle LBW in the region.

**Supplementary Information:**

The online version contains supplementary material available at 10.1186/s12889-021-10886-7.

## Background

Low birth weight (LBW) newborns (< 2500 g at birth) have higher risks of adverse health outcomes during childhood and adult life [1, 2], including increased risk of non-communicable disease as well as neurological and cognitive disorders [3–5]. Worldwide, around 15–20% (20 million infants, approximately) of all births are LBW, and almost 95% of them are born in low- and middle-income countries (LMICs) [6]. Although in Latin America LBW prevalence is low (around 8.7 in year 2015) compared to other developing regions [7], its rate of reduction has stagnated over the last 2 decades.

LBW has been linked to maternal, demographic, biologic and behavioral characteristics [8, 9] as well as to the socioeconomic and environmental characteristics of households and neighborhoods [10–13], and even to macro-economic conditions [14]. Previous studies suggested that mothers with low educational level [15] and those living in more disadvantaged neighborhoods are more likely to give birth to a LBW newborn [16]. In contrast, other work has found that within a country LBW rates are higher in areas with higher per capita income compared to those with lower per capita income [17].

Several mechanisms linking social factors to LBW have been proposed. One example focused on explaining high levels of LBW among African American women in the US is the “weathering hypothesis” that posits that high levels of social stressors resulting from long-term social disadvantage and racism lead to adverse pregnancy outcomes [18, 19]. Other mediating factors linking social deprivation to LBW include smoking, alcohol and drug consumption, psychosocial stress, low maternal body mass index or weight gain during pregnancy and insufficient prenatal care [20, 21]. In contrast, other mechanisms may lead to more LBW in wealthier areas. For example, wealthier contexts may have a higher prevalence of pregnancies at older maternal age, which are often associated with LBW. Additionally, the use of new health technologies in the preconception, prenatal, and perinatal periods [22] including the use of C-sections [23] may lead to an increase in the proportion of LBW, especially in higher socioeconomic groups, which have greater access to such procedures [15]. This is the so-called “epidemiological paradox of LBW” [3, 17, 24].

A separate body of work has examined urban-rural differences in LBW. In the U.S., both population-dense urban areas and more isolated rural regions had higher LBW compared to other regions [25, 26]. In other work in the U.S., urbanization had a protective effect on LBW [27]. Studies from Brazil reported a higher risk of LBW linked to greater urbanization [28, 29]. A number of factors including differences in maternal stress, smoking, alcohol and drug consumption during pregnancy [29–31] may underlie part of the urban-rural disparities in LBW. Access to new technologies may also play a role in the higher LBW observed in urban compared to rural areas in some studies. Differences in LBW between urban and rural areas appear to be heterogeneous in part because or large differences within urban areas. However, differences in LBW within urban areas and the factors associated with differences have not been investigated.

Latin America is one of the most urbanized and unequal regions in the world, with great heterogeneity in population socio-economic conditions, health infrastructure, and health outcomes [32, 33]. Understanding how social features are related to LBW in urban environments is important to the development of strategies aimed at reducing LBW in the region. To the best of our knowledge, these important aspects remain unexplored in Latin America. Based on unique data compiled by the SALURBAL project (Urban Health in Latin America; ‘Salud Urbana en América Latina’), the aims of this study were to quantify variability in LBW across cities in Latin America, and to evaluate the associations of socio-economic characteristics at various levels (maternal, sub-city and city) with the prevalence of LBW, while accounting for individual-level characteristics. We hypothesized that (1) LBW varies significantly within and between cities, (2) lower maternal education is associated with higher prevalence of LBW and that (3) worse city social environments, characterized by lower population educational attainment of sub-city units and by lower social environment score of cities, are associated with higher prevalence of LBW, independent of maternal education.

## Methods

### Data sources and sample

Data was compiled by the SALURBAL project, an interdisciplinary, multinational and collaborative initiative focused on characterizing the drivers of urban health and urban health inequalities across cities of the region [34]. Cities included in this study were those with a population of more than 100,000 people, in 8 countries: Argentina, Brazil, Chile, Colombia, Costa Rica, Guatemala, Mexico, and Peru. Cities were defined as agglomerations of administrative units (i.e., *municipios*, *comunas*, *partidos*, *delegaciones*, *cantones*, or *corregimientos*) that are covered, at least in part, by the urban extent of the city. Sub-cities were defined as administrative units nested within cities. In some cases, the city included a single sub-city unit, and in other cases, the city included multiple sub-city units [35].

Our study included data on more than 4.5 million births in 2014 from 360 cities and 1321 sub-cities (see Supplementary material, Table S1). Of the 4,690,190 live births occurring during 2014, 86,120 were excluded because they were missing data on birth weight. Further, we excluded 81,010 births with missing data on maternal age or education. The final sample included 4,531,699 births (see Supplementary material, Figure S1). Excluded births (*n* = 158,491) had higher LBW prevalence than those included (8.5% vs 7.8%), were more likely to have maternal education in the intermediate category, and to be births to mothers aged 19 years and younger, but differences between excluded and included births were small (see Supplementary material, Table S2).

### Outcome and maternal characteristics

Individual data on birth weight and mother’s sociodemographic characteristics for all live births came from the 2014 National Live Birth Registries of each country. LBW –defined as less than 2500 g at birth [6]– was used as the outcome. Maternal covariates available for each live birth included maternal age (years) (≤ 19, 20–24, 25–29, 30–34, ≥ 35) and maternal educational level (less than primary, at least primary but less than completed secondary, complete secondary or above). Both maternal age [36–39] and education [15, 40] are established predictors of LBW.

### Social environment

Sociodemographic characteristics of cities were retrieved from the closest available national population census in each country: 2017 (Peru), 2011 (Costa Rica), 2010 (Argentina, Brazil and Mexico), 2005 (Colombia), and 2002 (Chile and Guatemala). We used a Social environment index (SEI) at the city level [41] as a proxy of the contextual social environment of the cities. The city SEI was created by averaging the z-scores of four social indicators: percentage households with water connection in the dwelling, percentage households with connection to the sewage network, percentage overcrowded households (more than 3 people per room, inverted), and percentage individuals aged 25 or above with primary education completed or above. Overall, higher SEI corresponds to better social environment of the cities.

Additionally, we used a score of population educational attainment at the sub-city level [42] to capture within city heterogeneity in area socioeconomic status (SES). This indicator was obtained by adding the z-scores of the percentage of population age 25 or above that had completed high school level or above and the percentage of population age 25 or above that completed university level or above. Higher values correspond to higher population educational attainment of sub-cities. Sub-city education score and the city SEI were weakly correlated (*Spearman’s* r_s_ = 0.15; *p* <  0.001) and both were standardized.

### Other contextual variables

Analyses were adjusted for city population size and gross domestic product (GDP) at the city level as both could confound the association of SEI with LBW. City size may be related to health care access [15] and changes in GDP and economic contraction had been linked to birth outcomes [14]. Available GDP per capita data in SALURBAL (in constant 2011 international USD for year 2014) was derived from modelling approaches for larger administrative units attributed to cities [43]. City size was obtained from population projections for the year 2014. Both city population size and GDP of cities were standardized in regression analysis. Finally, we accounted for the impact of country level factors by including countries as fixed effects.

### Statistical analyses

We first described the proportion of LBW across cities by countries. Then, we calculated the variability in the sub-city prevalence of LBW (hypothesis 1) by using a three-level linear mixed model with percent LBW at the sub-city level as the outcome, and random intercepts for cities and countries. The random part allowed us to evaluate the variation in sub-city LBW within cities, between cities within countries, and between countries. We report the percentage of the variance at each level dividing each variance component by the total variance.

We also examined the distribution of individual and contextual characteristics for births with and without LBW. To test hypotheses 2 and 3 we fitted three-level mixed-effects Poisson models with cross-classified cells of maternal age and education nested within sub-cities, sub-cities nested within cities and country fixed effects. Final models were fitted as follows:

$$ \mathit{\log}\ \left({L}_{ijk}\right)={\gamma}_{000}+{\gamma}_{100}{E}_{ijk}+{\gamma}_{200}{A}_{ijk}+{\gamma}_{010}{U}_{jk}+{\gamma}_{001}{S}_k+{\gamma}_{002}{G}_k+{\gamma}_{003}{P}_k+{C}_c+{\vartheta}_{jk}+{\vartheta}_k+\mathit{\log}\ \left({N}_{ijk}\right) $$

where *L*_*ijk*_ is the number of LBW for cell *i* in sub-city *j* in city *k*; *N*_*ijk*_ is the overall number of live births in each cell; *γ*_100_ is the coefficient associated with maternal education; *γ*_200_ is the coefficient associated with maternal age; *γ*_010_ is the coefficient associated with sub-city population educational attainment; *γ*_001_ is the coefficient associated with city SEI; *γ*_002_ is the coefficient associated with cities GDP; *γ*_003_ is the coefficient associated with city population size of cities; *Cc* corresponds to the fixed effect of countries; *ϑ*_*jk*_ and *ϑ*_*k*_ are random effects for sub-cities and cities, respectively.

To test our hypotheses regarding associations of maternal education, sub-city SES and city social environment with LBW we fitted five models of increasing complexity. First, we estimated an empty model with no explanatory variables and random intercepts for sub-cities and cities. Model 1 added maternal age and education. Model 2 added sub-city population educational attainment (model 2). We then added city SEI as well a potential city-level confounders of city GDP and population size (model 3). Finally, model 4 added country fixed effects. We calculated the change in variance across models by using the proportional change in variance (PCV). PCV expresses the change in the proportion of the sub-city and city variance in a given model explained by adding specific variables in the subsequent model [44, 45]. All analyses were carried out in Stata 15.

## Results

**Fig. 1:**
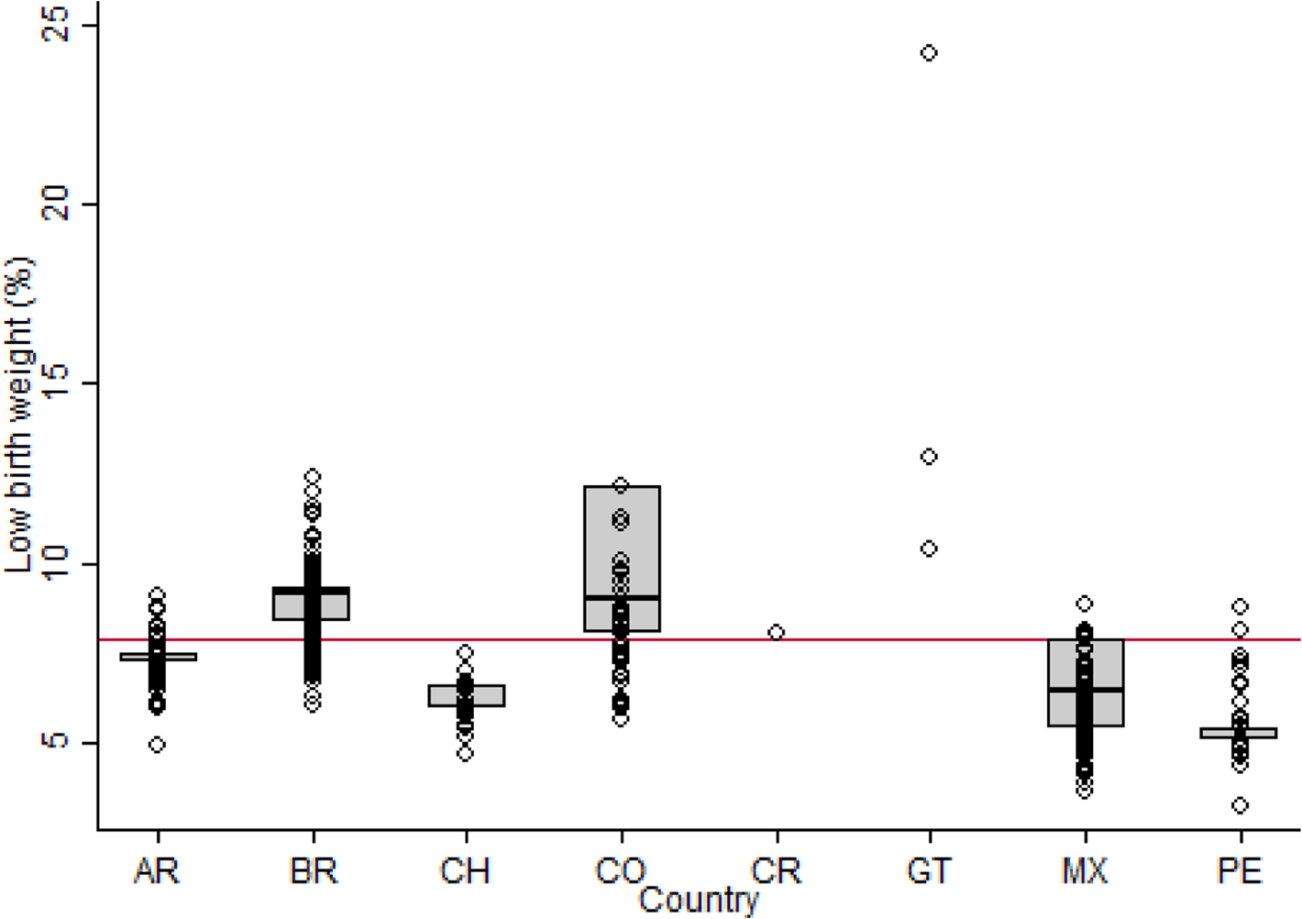
Prevalence of low birth weight (LBW; < 2500 g) in 360 cities from eight Latin American countries. Each dot represents the proportion of LBW among live births in cities. The red line indicates the median proportion of LBW in cities (7.8%) for the sample. Ref.: AR: Argentina, BR: Brazil, CH: Chile, CO: Colombia, CR: Costa Rica, GT: Guatemala, MX: Mexico, PE: Peru

Of the 360 cities in the sample, 42.2% were in Brazil, 25.6% in Mexico, 9.7% in Colombia, 9.2% in Argentina, 6.4% in Peru, 5.8% in Chile, 0.8% in Guatemala and 0.3% in Costa Rica (see Supplementary material, Table S1). The median number of live births per city was 34,029, ranging from a low of 753 to a high of 313,634. Figure 1 shows the prevalence of LBW in each city by country. The median LBW proportion across all cities was 7.8. Median prevalence by country ranged from a high of 13.0 in Guatemala to a low of 5.0 in Peru. Of the total LBW variability across sub-city units, 76% was between countries, 8% was between cities within a country, and 16% was within cities (see Supplementary material, Table S3). There was still substantial variability in LBW across cities within a country as illustrated in Fig. 1.

Table 1 shows individual and contextual characteristics of live births by LBW status. Low birth weight births were characterized by lower maternal education than normal birthweight births; specifically, we observed a higher proportion of mothers with less than primary education in LBW compared to normal birthweight births. Low birth weight births also had higher proportions of mothers under 19 years and over 35 years of age compared to normal weight births. Low birth weight births had higher mean sub-city educational attainment, lower mean city social environment index, lower mean city GDP, and higher mean city population compared to normal weight births, although differences were small (Table 1).

**Table 1:** Individual and contextual characteristics of live births by low birth weight status. Argentina, Brazil, Chile, Colombia, Costa Rica, Guatemala, Mexico and Peru; 2014

		Low birth weight (< 2500 g)		
	Overall	No	Yes	Comparison
	*n* = 4,531,699	*n* = 4,177,549	*n* = 354,150	(*t*-test or χ^2^) *p* value
Maternal education, % distribution				
Less than primary	9.3	9.2	11.2	< 0.001
At least primary but less than completed secondary	49.5	49.5	49.0	
Complete secondary and above	41.2	41.3	39.8	
Maternal age, % distribution				
≤ 19	16.5	16.4	18.1	< 0.001
20–24	26.2	26.4	23.9	
25–29	24.3	24.5	21.9	
30–34	19.9	19.8	20.0	
≥ 35	13.1	12.9	16.1	
City characteristics, mean (SD)				
Score of sub-city population educational attainment	0.166 (1.381)	0.164 (1.383)	0.189 (1.350)	< 0.001
Score of city social environment index	0.219 (0.518)	0.219 (0.517)	0.216 (0.528)	< 0.001
City gross domestic product, USD	18,325 (9717)	18,353 (9749)	17,989 (9325)	< 0.001
City population size, millions	5.9 (7.2)	5.9 (7.2)	6.2 (7.3)	< 0.001
Countries, % distribution				
Argentina	11.2	11.2	10.5	< 0.001
Brazil	38.1	37.6	43.4	
Chile	4.0	4.1	3.2	
Colombia	8.9	8.8	10.8	
Costa Rica	0.7	0.7	0.8	
Guatemala	1.6	1.5	2.8	
Mexico	29.1	29.5	24.2	
Peru	6.4	6.6	4.4	

Table 2 shows the estimated prevalence rate ratio (PRR) of LBW associated with maternal education, and social environment characteristics. Mothers with less than primary education had higher prevalence of LBW (PRR 1.12; 95% CI 1.10, 1.13) than those with complete secondary and above education (model 1). Maternal age showed a significant U-shaped association with LBW prevalence, with higher prevalence of LBW among mothers ≥35 (PRR 1.35; 95% CI 1.33, 1.36) and ≤ 19 years old (PRR 1.21; 95% CI 1.19, 1.22) compared to mothers aged 25–29 (model 1). A 1 SD higher score of sub-city population educational attainment was significantly associated with slightly higher prevalence of LBW (PRR 1.04; 95% CI 1.03, 1.04). There was no association between city SEI and LBW prevalence (PRR 1.01; 95% CI 0.98, 1.03) after adjusting for GDP and population size of cities (model 3). After including countries as fixed effects (model 4), a higher score of sub-city educational attainment remained associated with higher prevalence of LBW (PRR 1.04; 95% CI 1.03, 1.04), and higher city SEI (PRR 1.02; 95% CI 1.00, 1.04) became significantly associated with higher prevalence of LBW although the association was very small. The association of maternal education with LBW did not change substantially when sub-city and city social environment characteristics were adjusted for. Cities with larger populations had significantly higher prevalence of LBW in the fully adjusted model (PRR per 1 SD increase 1.06; 95% CI 1.01, 1.12). Peru had the lowest prevalence of LBW while Guatemala and Brazil (PRR vs Peru 3.11 (95% CI 2.59, 3.73) and 1.60 (95% CI 1.49, 1.71), respectively) had the highest LBW prevalence.

**Table 2:** Low birth weight prevalence rate ratios (PRR) associated with maternal sociodemographic and social environmental characteristics. Argentina, Brazil, Chile, Colombia, Costa Rica, Guatemala, Mexico and Peru; 2014

Cities (*n* = 360)	Low birth weight (< 2500 g)			
Sub-cities (*n* = 1321)	Model 1	Model 2	Model 3	Model 4
Cells (*n* = 19,375)	PRR (95% CI)			
Maternal age; years				
25–29	Ref.	Ref.	Ref.	Ref.
≤ 19	1.21 (1.19, 1.22)	1.21 (1.19, 1.22)	1.21 (1.19, 1.22)	1.21 (1.19, 1.22)
20–24	1.02 (1.01, 1.03)	1.02 (1.01, 1.03)	1.02 (1.01, 1.03)	1.02 (1.01, 1.03)
30–34	1.11 (1.09, 1.12)	1.11 (1.09, 1.12)	1.11 (1.09, 1.12)	1.10 (1.09, 1.12)
35+	1.35 (1.33, 1.36)	1.35 (1.33, 1.36)	1.35 (1.33, 1.36)	1.35 (1.33, 1.36)
Maternal education				
Completed Secondary and above	Ref.	Ref.	Ref.	Ref.
At least Primary; Less than completed Secondary	1.00 (0.99, 1.00)	1.00 (0.99, 1.01)	1.00 (0.99, 1.01)	1.00 (0.99, 1.00)
Less than Primary	1.12 (1.10, 1.13)	1.12 (1.11, 1.13)	1.12 (1.11, 1.13)	1.12 (1.10, 1.13)
Sub-city educational attainment, z-score	–	1.04 (1.03, 1.04)	1.04 (1.03, 1.04)	1.04 (1.03, 1.04)
City social environment index, z-score	–	–	1.01 (0.98, 1.03)	1.02 (1.00, 1.04)
City gross domestic product, z-score			0.99 (0.97, 1.01)	0.99 (0.98, 1.01)
City population size, z-score			1.08 (0.99, 1.17)	1.06 (1.01, 1.12)
Countries				
Peru	Ref.	Ref.	Ref.	Ref.
Argentina	–	–	–	1.37 (1.26, 1.49)
Brazil	–	–	–	1.60 (1.49, 1.71)
Chile	–	–	–	1.13 (1.02, 1.25)
Colombia	–	–	–	1.51 (1.39, 1.64)
Costa Rica	–	–	–	1.42 (1.08, 1.86)
Guatemala	–	–	–	3.11 (2.59, 3.73)
Mexico	–	–	–	1.10 (1.02, 1.18)
*Random parameters*				
City variance intercept (std. error)	0.054 (0.005)	0.056 (0.005)	0.055 (0.005)	0.018 (0.002)
Sub city variance intercept (std. error)	0.007 (0.001)	0.006 (0.001)	0.006 (0.001)	0.005 (0.001)
PCV (city)	5.3^a^	+ 3.7	1.8	67.3
PCV (sub-city)	12.5^b^	14.3	0.0	16.7

Cells of LBW aggregated by maternal education and maternal age nested within sub-cities and within cities. Outcome: LBW (< 2500 g) reported as prevalence rate ratios (PRR) considering counts of LBW and total births (offset). Empty model includes random intercepts for sub cities and cities (not shown); model 1 includes maternal age and maternal education; model 2 adds sub-city population educational attainment to model 1; model 3 adds city social environment index, gross domestic product and population size of cities to model 2; model 4 adds countries as fixed effects. PCV: Proportional change in variance compared to preceding model. The positive sign indicates and increase in variance; ^a^ Calculated based by comparing to city variance intercept (std. error) of the empty model (0.057 (0.005); not shown); ^b^ Calculated based by comparing to sub-city variance intercept (std. error) of the empty model (0.008 (0.001); not shown)

The addition of sub-city and city characteristics did not substantially reduce the within or between city variance. However, the inclusion of countries in the model substantially reduced the city and sub-city variance (67.3 and 16.7% respectively) observed in model 3.

## Discussion

We examined variability of LBW prevalence across countries, cities and sub-cities and the associations of LBW with maternal, sub-city and city socioeconomic characteristics in 360 cities from 8 countries in Latin America. We found considerable heterogeneity in the distribution of LBW across cities and sub-cities. We also found that low maternal education was associated with higher prevalence of LBW. In contrast, higher sub-city education and better city social environment were independently associated with higher LBW prevalence after adjustment for maternal characteristics, city size and city GDP.

Other work has explored LBW variability at sub-national levels in the U.S. [46–49], Ireland [50] and Brazil [29, 51], but few studies have investigated variability in LBW across large samples of diverse urban areas. We found that most of the variability in LBW was between countries. However, there was also substantial variability across cities within countries and across sub-city units within cities.

We found a higher prevalence of LBW among mothers in the lowest category of educational attainment (less than primary school). This is consistent with our hypothesis, with prior work in high-income countries [52, 53] and with a meta-analysis including high-, upper-middle and middle-income countries, in which high maternal education showed a protective effect against LBW, whereas medium degree of education had no protection when compared to low maternal education [15]. In Brazil, improvements in maternal education and antenatal care coverage reduced the risk for LBW [54]. In addition, Silvestrin et al. [55] reported a significant decrease over time in mean birth weight in neonates born to Brazilian mothers with higher educational attainment. The association between maternal education and LBW may be related to behavioral factors before and during pregnancy such as stress, smoking, alcohol and drug consumption [55]. Differences in access to prenatal care and treatment of conditions like maternal hypertension may also differ across education groups [56, 57]. Other research has linked the use of health technologies in the prenatal period to an increasing proportion of LBW in higher socioeconomic groups [22]. Thus, the patterns that we see by maternal education may reflect countervailing influences.

In contrast to our hypothesis, higher sub-city education was associated with higher prevalence of LBW. Although the association was not very strong, it is still notable for its consistency across models. A large body of work mostly from higher income countries has shown that higher neighborhood SES is associated with lower prevalence of LBW [16, 58, 59]. However, evidence for larger areas like counties is more mixed [46]. Our results are aligned with studies describing higher risk of LBW in areas with better social environments. Better socioeconomic contexts of municipalities were associated with higher risk of LBW in Colombia [3]. Young et al. [60] did not find associations of birth weight with community social environment in Massachusetts. Community levels of unemployment were not associated with LBW in Toronto, but were positively associated with LBW in Baltimore, Boston, Chicago and Philadelphia [46]. Similarly, and also contrary to our hypotheses, we found that a summary city-level indicator of socioeconomic development was weakly positively associated with LBW even after sub-city and maternal characteristics were taken into account. Our results are consistent with other studies in Brazil where better socioeconomic contexts of cities [17] and regions [24] were associated with higher rates of LBW. While we found no association between city GDP and LBW, there was higher prevalence of LBW among more populated cities.

Several factors could explain the slightly higher prevalence of LBW that we observed in cities with higher SEI and in sub-city areas with higher education. These include factors occurring to a higher extent in wealthier areas such as late pregnancies [15] (although our analyses adjust for maternal age) or the increased use of health technologies such as assisted reproductive technology [22]. Indeed, the use of assisted reproductive technology was related to LBW and C-sections [61], and since elective C-sections are more common in wealthier areas and are usually planned earlier in gestation, there may exist a link between these and late-preterm live births [23, 62]. Other mechanisms could include better access to health care and greater medical interventions that allows pre-term newborns to survive even at extreme gestational ages. In addition, the under-registration of live births and registration of livebirths as stillbirths in poorer areas may explain why LBW rates in Brazil are higher in richer than in poorer municipalities [17]. Of note, larger cities also tended to have higher rates of LBW. Mechanisms could include increasing exposure to environmental hazards that are associated with LBW and prematurity such as air pollution as well as the health care use and access related processes discussed previously.

We controlled for country effects in order to account for unobserved country heterogeneity related to the possible impact of health care policies, cultural features around maternity and childcare, and fertility levels, among other factors. Although Latin America have experienced a decrease in fertility rate across all age groups, adolescent birth rates still among the highest in the world. We assumed that adjusting for maternal age would help to partially address this issue. Our results showing substantial variability in LBW across countries as well as significant associations of country fixed effects with LBW suggest that country factors deserve additional exploration. However, controlling for countries in the fully adjusted model (model 4) did not substantially change associations of other SES features with LBW. In addition, variability in LBW across cities persisted even after maternal education and age as well as sub-city and city social environment factors were taken into account, suggesting that other city factors may be important to LBW. Additional research is needed in LMICs on how features of urban environments vary across cities and also across communities or neighborhoods.

### Limitations and strengths

An important limitation of our analyses is that we combined all birthweights under 2500 g which includes a diverse population of live births, from pre-term newborns with adequate birthweight for their gestational age to term newborns that are small for their gestational age (intrauterine growth retardation). Data on gestational age was not available for all countries, and consequently, we were unable to differentiate between these two groups, which have different etiologies and could be differentially related to the factors we studied. The separation of pre-term from small for gestational age births would allow better exploration of the mechanisms that might be involved in generating the patterns that we describe. However, LBW remains a meaningful health outcome in its own right and is often an important metric tracked in perinatal health. An additional limitation is that social environment characteristics retrieved from censuses were at different years and not always consistently aligned with the years for which birth outcomes were obtained. We therefore assumed that social environment measures were relatively stable across the years examined. The use of sub-city units, although capturing some heterogeneity within cities, does not capture neighborhood factors that may also be important in LBW. In addition, there are other important proximal determinants of LBW at both individual or aggregated levels that we were not able to account for due to lack of data. These include important risk factors for LBW such as inadequate or insufficient antenatal care [63, 64] and low maternal dietary diversity [65]. We also did not include information on fertility at the city and sub-city level.

In spite of the efforts to reach universal registration, undercounting of births is still an issue in the region (nearly 6% of children under 5 years old have not had their births registered in Latin America and the Caribbean) [66]. Although the coverage of vital statistics registration is better in urban settings compared to rural areas, the undercount of births tends to be higher in areas with lower socioeconomic status [66, 67]. If undercounted births in lower socioeconomic areas are more likely to have LBW, our estimates of area associations of SES with LBW may be biased. Similarly, misclassification of birthweight is also possible. The capacity to accurately measure some variables such as LBW in resource-poor countries is a well-documented challenge [68].

Major strengths of our study include the large and unique dataset of millions of live births across 8 countries, 360 cities, and the linkage to harmonized maternal, sub-city and city level factors. The multilevel structure allowed us to assess LBW variability across a great number of sub-city units (1321) and city units in different countries, and to simultaneously examine maternal, sub-city and city characteristics. Overall, our study provides a singular perspective on the drivers of LBW within urban social environments in Latin America.

In summary, we found that better social environments at both city and sub-city levels, as well as low maternal education were associated with higher prevalence of LBW newborns, independent of maternal age and other features of the social environment of cities like population size and GDP. Furthermore, we found substantial variations in LBW across countries and to a lesser extent across cities within countries and across sub-cities within cities. Our findings add evidence on the relevance of socioeconomic characteristics at both the individual and contextual levels for LBW, based on a large number of Latin American cities.

The increasing inequities in health within urban contexts together with the persistent rates of LBW worldwide, highlight the need for public health strategies to prevent LBW in the growing cities of lower- and middle-income countries. Urban health studies exploring the impact of urban environments on health in the global south have recently received increasing attention [34, 69]. Our results highlight the importance of local and broader social environments in shaping LBW in urban settings of Latin America, and suggest that interventions focused on improving maternal education may be useful in reducing LBW in the region.

## Supplementary Information

**Additional file 1: Figure S1**. Flow chart describing the sample selection involving eight Latin American countries (Argentina, Brazil, Chile, Colombia, Costa Rica, Guatemala, Mexico, Peru) for year 2014. **Table S1**. Study characteristics by country. **Table S2**. Characteristics of included and excluded cases. **Table S3**. Variance components for sub-city prevalence of low birth weight in eight Latin American countries (Argentina, Brazil, Chile, Colombia, Costa Rica, Guatemala, Mexico and Peru) for year 2014.

## Data Availability

When the data can be made public without violating confidentiality -once the SALURBAL project ends-, it will be placed in a public repository as required by the funder (Wellcome Trust). The SALURBAL project welcomes queries from anyone interested in learning more about its dataset and potential access to data. To learn more about SALURBAL’s dataset, visit https://drexel.edu/lac/ or contact the project at salurbal@drexel.edu.

## Abbreviations

GDP: Gross domestic product
LBW: Low birth weight
LMICs: Low- and middle-income countries
PCV: Proportional change in variance
SALURBAL: Salud Urbana en América Latina
SEI: Social environment index
SES: Socioeconomic status
