# Supplementary material for: Urban social environment and low birth weight in 360 Latin American cities
Source: BMC Public Health. 2021 Apr 26;21:795. doi: 10.1186/s12889-021-10886-7 (PMC8073945; doi:10.1186/s12889-021-10886-7)
Supplement: Supplementary file 1 — Additional file 1: Figure S1. Flow chart describing the sample selection involving eight Latin American countries (Argentina, Brazil, Chile, Colombia, Costa Rica, Guatemala, Mexico, Peru) for year 2014. Table S1. Study characteristics by country. Table S2. Characteristics of included and excluded cases. Table S3. Variance components for sub-city prevalence of low birth weight in eight Latin American countries (Argentina, Brazil, Chile, Colombia, Costa Rica, Guatemala, Mexico and Peru) for year 2014. [file 12889_2021_10886_MOESM1_ESM.pdf]

SUPPLEMENTARY MATERIAL

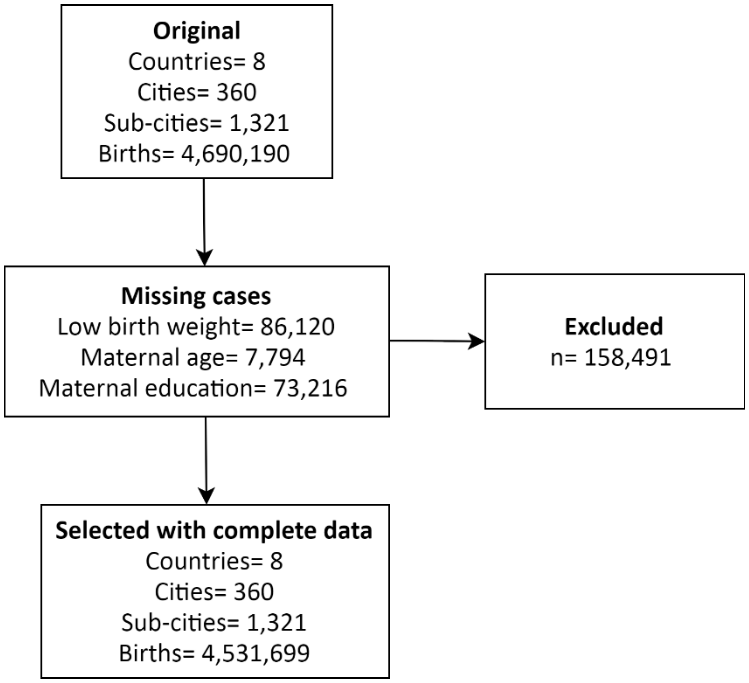

Figure S1. Flow chart describing the sample selection involving eight Latin American countries (Argentina, Brazil, Chile, Colombia, Costa Rica, Guatemala, Mexico, Peru) for year 2014.

Table S1. Study characteristics by country.

Country	Live births n (%)	Sub-cities n (%)	Cities n (%)	Sub-city population-level educational attainment Median (IQR)	City social environment index Median (IQR)
Argentina	506,291 (11.2)	110 (8.3)	33 (9.2)	-0.56 (1.36)	0.32 (0.05)
Brazil	1,726,288 (38.1)	422 (32.0)	152 (42.2)	0.32 (1.77)	0.28 (0.56)
Chile	180,534 (4.0)	81 (6.1)	21 (5.8)	-0.68 (1.00)	1.04 (0.18)
Colombia	404,286 (8.9)	84 (6.4)	35 (9.7)	0.11 (1.69)	0.60 (0.59)
Costa Rica	32,820 (0.7)	29 (2.2)	1 (0.3)	1.38 (1.78)	0.53 (0.00)
Guatemala	72,761 (1.6)	20 (1.5)	3 (0.8)	-0.74 (2.27)	-1.04 (0.00)
Mexico	1,316,954 (29.1)	406 (30.7)	92 (25.6)	-0.14 (1.56)	0.32 (0.65)
Peru	291,765 (6.4)	169 (12.8)	23 (6.4)	1.97 (1.86)	0.47 (0.17)

Live births correspond to year 2014; Sub-city population-level educational attainment: higher values correspond to higher population educational attainment of sub cities; City social environment index: higher values indicate better social environment of cities; IQR, interquartile range.

Table S2. Characteristics of included and excluded cases.

	Overall n= 4,690,190	Included n= 4,531,299	Excluded [a] n= 158,491	Comparison (t or χ^2) p value
Low birth weight, (< 2,500 g), % distribution	7.8	7.8	8.5	< 0.001
Maternal education, % distribution				
Complete secondary and above	41.1	41.2	36.7	< 0.001
At least primary but less than completed secondary	49.6	49.5	57.1	
Less than primary	9.3	9.3	6.2	
Maternal age, years , % distribution				
≤ 19	16.6	16.5	19.8	< 0.001
20-24	26.2	26.2	28.7	
25-29	24.2	24.3	22.8	
30-34	19.8	19.9	17.1	
≥ 35	13.1	13.1	11.6	
Score of sub-city population educational attainment, mean (SD)	0.152 (1.377)	0.166 (1.381)	-0.244 (1.210)	< 0.001
Score of city social environment index, mean (SD)	0.216 (0.519)	0.219 (0.518)	0.114 (0.556)	< 0.001

Countries, % distribution				
Argentina	11.4	11.2	18.2	< 0.001
Brazil	37.3	38.1	13.3	
Chile	3.9	4.0	0.3	
Colombia	8.9	8.9	6.7	
Costa Rica	0.7	0.7	0.6	
Guatemala	1.6	1.6	1.2	
Mexico	30.1	29.1	59.3	
Peru	6.2	6.4	0.4	

[a] Excluded cases were those with missing data on any of the following characteristics: birth weight (n= 86,120), maternal age (n= 7,794), or maternal education (n= 73,216).

Table S3. Variance components for sub-city prevalence of low birth weight in eight Latin American countries (Argentina, Brazil, Chile, Colombia, Costa Rica, Guatemala, Mexico and Peru) for year 2014.

Countries (n= 8)	Sub-city low birth weight (LBW, < 2,500 g), %
Cities (n= 360)	Empty model
Sub-cities (n= 1,321)	
Random parameters	
Country variance (std. Error)	9.797 (5.116)
City variance (std. Error)	1.019 (0.132)
Sub-city variance (std. Error)	2.021 (0.088)

Multilevel linear model: sub-cities nested within cities, nested within countries; outcome: percentage of LBW (< 2,500 g) in sub-cities.
